# Neighbourhood deprivation and obesity among 5656 pre-school children—findings from mandatory school enrollment examinations

**DOI:** 10.1007/s00431-021-03988-2

**Published:** 2021-02-11

**Authors:** Thuy Ha Nguyen, Simon Götz, Katharina Kreffter, Stefanie Lisak-Wahl, Nico Dragano, Simone Weyers

**Affiliations:** 1 Faculty of Medicine, University Hospital Duesseldorf, Centre for Health and Society, Institute of Medical Sociology, Moorenstrasse 5, 40225 Duesseldorf, Germany; 2 Akademie für Öffentliches Gesundheitswesen, Düsseldorf, Germany

**Keywords:** Paediatric obesity, Environmental health, Child health, Social inequalities, School entrance

## Abstract

The risk of child obesity is strongly related to socioeconomic factors such as individual socioeconomic position (SEP) and neighbourhood deprivation. The present study analyses whether the relationship between neighbourhood deprivation and child obesity differs by child’s individual SEP. Data from 5656 children (5–7 years) from the mandatory school enrollment examinations of the pre-school cohorts 2017/2018 in Düsseldorf were analysed. Obesity was determined by the age- and gender-specific body mass index (BMI); neighbourhood deprivation by using the socio-spatial degree of deprivation of the children’s residential addresses; and individual SEP by the level of parental education. Using Poisson regression, we estimated prevalence ratios (PR with 95% confidence interval (CI)) of child obesity by neighbourhood deprivation and parental education. Interactions between neighbourhood deprivation and parental education were tested. The prevalence of child obesity increases with the degree of neighbourhood deprivation. Compared to children living in low deprivation neighbourhoods, the proportion of obese children was twice as high in high deprivation neighbourhoods (PR=2.02; CI=1.46–2.78). Likewise, children from families with medium and low education have twice the risk for obesity compared to children with high parental education (PR=2.05; CI=1.46–2.78). The relationship between neighbourhood deprivation and child obesity was significantly moderated by parental education; it was stronger for higher parental education than for medium and low parental education (*p*<.001).

*Conclusion*: Our findings suggest that children from deprived neighbourhoods and families with lower education have a higher risk for child obesity. The identification of particularly deprived neighbourhoods with structural interventions in combination with the strengthening of parental health literacy seems reasonable.

**Table:** 

**What is Known:** *• Studies show that children from disadvantaged neighbourhoods are more frequently obese.*	
**What is New:** *• The relationship between neighbourhood deprivation and child obesity is significantly moderated by parental education. It is stronger for children with higher parental education than for children with medium and low parental education.*

**What is Known:**

*• Studies show that children from disadvantaged neighbourhoods are more frequently obese.*

**What is New:**

*• The relationship between neighbourhood deprivation and child obesity is significantly moderated by parental education. It is stronger for children with higher parental education than for children with medium and low parental education.*

## Introduction

About every fifth child in Europe is overweight or obese [2]. Although the prevalence of overweight and obesity has plateaued [1], a considerable percentage of children is affected.

Overweight and obesity in childhood increase the risk for obesity in adulthood [42] and chronic diseases such as type 2 diabetes and coronary heart disease [34]. Mental health and emotional wellbeing can be compromised by obesity and the experience of stigma [30, 31]. The World Health Organization has identified child obesity as one of the “most serious public health challenges of the 21st century” [16].

Identifying risk factors is crucial to developing preventive measures. Social ecological models of child obesity contain individual and environmental factors [27] including neighbourhood. In addition to the neighbourhood’s physical and social environments, its socioeconomic position (SEP) plays a role. Neighbourhood SEP influences the spatial and social environment (e.g. walkability, organised programmes, safety), which, in turn, affects the individual situation (e.g. health behaviour, utilisation, stress) and, as a result, child body weight [10, 13, 44].

Although pre-school years are formative for the development of health behaviours, only a few studies investigate the impact of neighbourhood SEP on child obesity in this age group. These studies show that children from disadvantaged neighbourhoods are more frequently obese [7–9, 12, 24, 37, 39]. However, the effect of the neighbourhood is attenuated after the individual SEP is taken into account [8, 12–14, 37, 43]. Instead of continuing to investigate if neighbourhood deprivation influences child obesity, attention should be turned to focus on the conditions under which this is relevant [22, 26] and whether a high individual SEP might be protective in high deprived neighbourhoods. From a salutogenic perspective, it could be argued that a higher SEP with more psychosocial and financial resources for health promoting behaviour mitigates the damaging effect of neighbourhood deprivation. Methodically, this leads to the question whether individual socioeconomic characteristics moderate the influence of the neighbourhood on child health and development [24]. The only study known to us that takes up this issue shows that higher individual-level income was protective for children living in low deprived neighbourhoods, but not for children who lived in high deprived areas [33].

A further limitation is that only a small number of studies used large-scale representative samples. They analysed the influence of neighbourhood on child obesity in the context of school enrollment medical screenings [8, 9, 12, 37]. These offer the advantages of large samples and the participation of families from all social positions resulting in little selection bias [42]. However, the interaction of neighbourhood and individual SEP was not addressed in these studies.

Seizing upon these limitations, this study aims to investigate in a large and representative sample whether the relationship between neighbourhood deprivation and child obesity differs according to the child’s individual SEP. Our hypothesis is that high individual SEP mitigates the effect of neighbourhood deprivation on child obesity. Understanding this association is important for effective policy initiatives to reduce child obesity disparities [13].

## Methods

Our cross-sectional study is based on the school enrollment medical screening of the pre-school children cohorts (5–7 years) 2017 and 2018 in Duesseldorf, Germany. This examination was conducted by the municipal health authorities and supplemented by us with a standardised paper-and-pencil parental questionnaire [42]. A proband identification number was assigned to every participant linking data of the medical screening, the parental survey (e.g. education, income and occupation) and the social area code. A response rate of 66% allowed the inclusion of 6480 cases in the study. We excluded 824 cases with missing values for the analysed variables, so that 5656 children remained in the sample (52% male). The excluded group has a higher prevalence of obesity (*χ*^2^=19.98; *p*>.001), has a higher proportion of low and medium educated parents (*χ*^2^=350.72; *p*>.001) and lives more often in deprived neighbourhoods (*χ*^2^=150.70; *p*>.001) as compared to the included group. The method was approved by the ethics committee and complied with the principles of the Declaration of Helsinki (Study no. 5664).

Obesity was identified based on the age- and gender-specific body mass index (BMI). It was calculated using child’s height, weight, sex and age which were objectively measured by the municipal health authorities. According to the gender- and age-specific percentiles of child BMI according to Kromeyer-Hauschild et al. [15], a child in the 90th percentile or above was classified as overweight and a child in the 97th percentile or above as obese. Overweight and obesity were combined into one category in the analyses and compared to the other children.

Neighbourhood deprivation was defined using the socio-spatial degree of deprivation for children’s residential addresses. The classification of the deprivation degrees was accomplished by the local authorities. Indicators like “welfare benefits”, “unemployment” and “living space per person” classify 166 social spaces into five neighbourhood types ranging from “very low” to “very high” [18]. The sample size for the category “very high” (*n*=306) was deemed too small for the analysis, so the categories “high” and “very high” were combined (*n*=1,401); this resulted in four categories with “very low degree of deprivation” as reference category.

The individual SEP was measured using the level of parental education. Following the CASMIN (“Comparative Analysis of Social Mobility in Industrial Nations”) classification [19], the highest general education diploma was combined with the highest level of vocational training for each parent. For parents with different levels of education, the higher level was selected. We wanted to compare the effect of neighbourhood deprivation on child obesity by individual SEP. Therefore, we used parental education as a binary variable to compare families with medium and low levels of education (at most the qualification to study at a university of applied science/university-track secondary school diploma with vocational training, CASMIN classification for the German educational system: 1a-2c_voc) with those who had a higher level of education (graduation from a university of applied science or a university, 3a–b).

Relevant covariates [17] are as follows: Parents’ employment: if both parents were not working at the time of the survey, they were considered unemployed. The reference category was families in which at least one parent worked full-time or part-time. Family status: if the child lived with only one parent, that parent was classified as a single parent family. Families in which both parents lived together served as reference category. Migration background: both parents were asked about their place of birth. If at least one parent had not been born in Germany, then a migration background was assumed. Families in which both parents had been born in Germany served as reference category.

### Data analysis

**Fig. 1:**
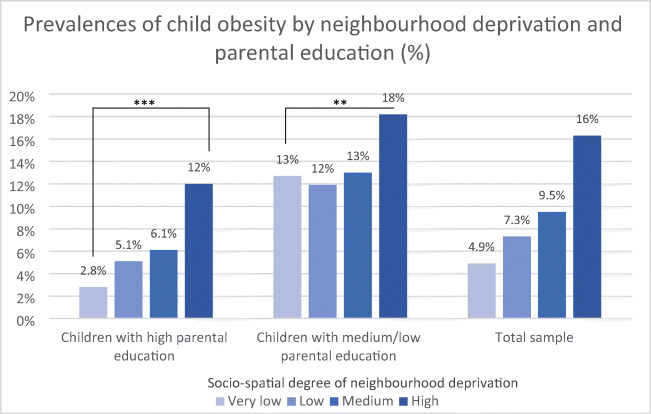
Prevalences of child obesity by neighbourhood deprivation and parental education in percentages (%). Asterisks represent statistical significance (* *p*<.05; ** *p*<.01; *** *p*<.001)

The absolute and relative frequencies of child obesity were described according to neighbourhood deprivation for the entire sample and stratified according to parental education (Fig. 1). Chi-square tests were used to compare categorical variables between girls and boys or medium/low and high parental education. Multicollinearity analysis of all variables showed acceptable values of the variance inflation factors (VIFs) ranging from 1.0 to 1.2 (results not shown). Then, we used Poisson regression with robust standard errors to estimate adjusted prevalence ratios (PR) of child obesity by neighbourhood deprivation and parental education. We calculated PRs instead of odds ratios because they provide less biased estimates [3]. Model 1 shows the crude model. We adjusted for age, gender, parental employment status, family status and migration background (model 2). To analyse if the association between neighbourhood deprivation and obesity differs by parental education, we tested for interactions and included interaction terms (model 3). To compare both models without and with interactions, we used a Wald test to assess if the interactions significantly increased the model fit. All analyses were conducted using Stata 14.

## Results

Table 1 shows the sample characteristics. 9.7% of the children were overweight or obese. There were no differences between boys and girls (*χ*^2^=0.06; *p*=.805). Twenty-eight percent and 25% of the children lived in neighbourhoods with medium and high deprivation, respectively. A total of 44% of the children lived in households with medium to low parental education.

**Table 1:** Sample description: observations (*n*) and percentages (%) or mean and standard deviation (SD)

Sample characteristic^1^	Categories	n	%
Gender	Male	2918	51.6
	Female	2738	48.4
Age, years [mean (SD)]		5.95	(0.27)
Obesity	No	5106	90.3
	Yes	550	9.7
Neighbourhood deprivation	Very low	995	17.6
	Low	1652	29.2
	Medium	1608	28.4
	High	1401	24.8
Parental education	High	3148	55.7
	Medium/low	2508	44.3
Employment status	At least one parent in employment	5303	93.8
	Both parents are not employed	353	6.2
Family status	Dual-parent families	4,953	87.6
	Single parents	703	12.4
Migration background	No	2946	52.1
	Yes	2710	47.9
Total		5656	100.00

^1^Variable distributions are reported as n and % unless otherwise specified

Overall, the percentage of obese children increases with the degree of deprivation from 4.9 to 16% (*p*<.001). Figure 1 shows the percentage of obese children according to neighbourhood deprivation and parental education. The differences in the prevalences between very low and high deprived neighbourhoods were higher for children with higher educated parents (2.8% vs. 12%; *p*<.001) than for children with medium or low educated parents (13% vs. 18%; *p*=.002). Furthermore, children from families with high education were less often obese than children from lower educated families (*p*<.001). This was observed in each stratum of neighbourhood deprivation; however, the gap between these numbers closes with increasing neighbourhood deprivation: The differences between both educational groups range from 10.2% in the least deprived neighbourhood (*χ*^2^=0.87; *p*=.349)and 6.9% in the low (*χ*^2^=12.38; *p*>.001) and medium deprived neighbourhoods (*χ*^2^=16.49; *p*>.001) to 6% in the most deprived neighbourhood (*χ*^2^=3.04; *p*=.081).

The adjusted PRs from the Poisson regression in Table 2 (model 2) confirm the results described above: The more deprived a neighbourhood is, the higher the prevalence of obese children. Compared with the reference group in a low deprived neighbourhood, the probability of obesity was twice as high in a highly deprived neighbourhood (PR = 2.02; CI = 1.46–2.78). Likewise, parental education had an influence on obesity: Children from families with medium and low education have a probability for obesity that is twice as high compared to children from families with high education (PR = 2.05; CI = 1.46–2.78). It should be noted that the PRs are not directly comparable across the models because they were calculated on different baselines [41]. The main effects from model 1 and 2 show the relationship of child obesity for parental education and neighbourhood deprivation, respectively, without taking the other independent variable into account, whereas the main effects in the first block of model 3 refer to the “high parental education” group only. In this group, high neighbourhood deprivation increases the probability of child obesity by 3.59 (CI = 2.20–5.87). The main effects in the second block in model 3 refer to the “very low neighbourhood deprivation” group only. In this group, medium/low parental education increases the probability of child obesity by 4.21 (CI=2.44–7.24). The interaction terms (neighbourhood deprivation x parental education) in block 3 in model 3 show that parental education significantly moderates the association between child obesity and neighbourhood deprivation. A Wald test confirms the significance (*χ*^2^=112.62, *p*<.001).

**Table 2:** Prevalence ratios with 95% confidence intervals for child obesity; results of Poisson regression models (*n*=5656)

	Model 1			Model 2			Model 3		
	PR	CI (95%)	p	PR	CI (95%)	p	PR	CI (95%)	*p*
Very low neighbourhood deprivation	Reference			Reference			Reference		
Low neighbourhood deprivation	1.34	0.97–1.85	.074	1.32	0.96–1.83	.087	1.77α	1.09–2.86	.021
Medium neighbourhood deprivation	1.54	1.12–2.12	.008	1.41	1.02–1.94	.036	2.00α	1.22–3.26	.006
High neighbourhood deprivation	2.29	1.66–3.15	<.001	2.02	1.46–2.78	<.001	3.59α	2.20–5.87	<.001
High parental education	Reference			Reference			Reference		
Medium/low parental education	2.13	1.77–2.58	<.001	2.05	1.69–2.47	<.001	4.21β	2.44–7.24	<.001
Low deprivation x medium/low parental education							0.53	0.28–1.01	.053
Medium deprivation x medium/low parental education							0.48	0.25–0.89	.021
High deprivation x medium/low parental education							0.36	0.20–0.67	.001
Pseudo *R*^2^		0.043			0.053			0.056	

*PR* prevalence ratio, *CI* confidence interval, *p* p-value, *Pseudo R*^*2*^ McFadden’s pseudo-R squared value. Model 1 unadjusted. Model 2 adjusted for age, child gender, employment status, family status and migration background. Model 3 adds interaction terms. α Main effects refer to the “high parental education” group only. β Main effects refer to the “very low neighbourhood deprivation” group only. Wald test for interaction: *χ*^2^=112.62, *p*<.001)

**Fig. 2:**
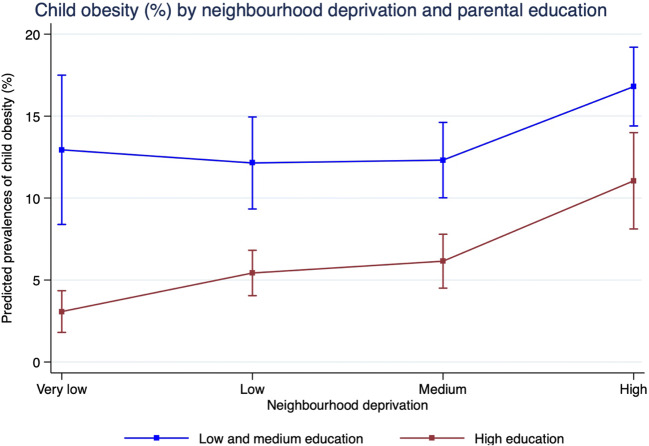
Predicted prevalences (%) of child obesity by neighbourhood deprivation and parental education based on margins calculated in Poisson regression with 95% confidence intervals. Predicted prevalences are adjusted for age, gender, employment status, family status and migration background

The results are further illustrated in Fig. 2 based on the calculation of predicted prevalences in the adjusted model 3 with interaction terms. It can be seen that the predicted prevalences increase less sharply with neighbourhood deprivation in the group of children with medium/low parental education (very low: 13% vs. high: 17%) than in the group of children with high parental education (3.0% vs. 11%). The association between neighbourhood deprivation and child obesity was stronger for higher parental education than for medium and low parental education. For children with high parental education, the prevalence remains low in low deprived neighbourhoods but increases with the degree of neighbourhood deprivation, whereas children with medium and low parental education have a higher prevalence rate throughout all neighbourhoods. However, the difference between both education levels becomes small in highly deprived neighbourhoods.

## Discussion

It was observed that the percentage of obese children increases with neighbourhood deprivation. However, this increase is stronger for children with higher parental education than for those with medium or low parental education and to such a degree that the difference between education levels in highly deprived neighbourhoods becomes small. Moreover, in all neighbourhoods, children of lower educated parents are more frequently obese than children of higher educated parents. It was also seen that there was a stronger effect of neighbourhood deprivation for children with high parental education than for children with medium or low parental education.

The relationship between neighbourhood deprivation and child obesity confirms previous evidence [14, 20, 36]. The association revealed between parental education and child obesity is also comparable to the previous study results [20, 27, 36]. In the study by Igel et al. [12], however, education (here: maternal education) had no significant influence on the prevalence of obesity.

There is currently little evidence regarding the question whether the association between neighbourhood deprivation and child obesity differs according to individual SEP. The only study known to us by Rossen [33] makes the same finding that the relationship between neighbourhood deprivation and obesity in children with higher parental income is stronger than in children with lower parental income. The finding is thus stable across two different SEP indicators and samples; despite this, it is counterintuitive. From a salutogenic perspective, we argued that education mitigates the damaging effect of neighbourhood deprivation. Therefore we hypothesised a flatter gradient along neighbourhood deprivation for the highly educated group, but it is, by contrast, steeper. This could be explained by the low prevalence of obesity in children of parents with a high education in a good neighbourhood. In other words, the baseline value is especially low. Conversely, the children whose parents have a medium and low education are more frequently obese even in good neighbourhoods. This speaks for the relevance of parental education regarding child obesity, independent of the environment.

Nevertheless, for both groups, the environment is relevant for child obesity. It is possible to distinguish between two mechanisms: (i) causation and (ii) selection. (i) On one hand, it can be assumed that neighbourhood deprivation favours an obesogenic environment [35]. For instance, high traffic density, a lack of parks and playgrounds and an inadequate infrastructure for local sports clubs limit the options for physical activity. Obesogenic environments are particularly relevant for children since they are restricted in their mobility and constantly embedded in their environment [35]. (ii) On the other hand, selection mechanisms could also be taking place. In this study, the high percentage of obese children of parents with high education in deprived neighbourhoods is striking. This could be due to downward social mobility, where highly educated parents move into socioeconomically disadvantaged neighbourhoods, e.g. as the result of separation or divorce. In a review [23], long maternal working hours and a permissive parenting style in high SEP families were risk factors for child obesity—factors that could occur more frequently in single parent households.

However, scrutinising the group of parents with high education in deprived neighbourhoods in our sample, it was observed that this frequently involved families with migration backgrounds (86%). A study by Renzaho et al. [32] shows that this group has a lower level of obesity literacy and is subject to cultural influences affecting body image and eating habits that increase the risk of child obesity. Simultaneously, there are barriers to participation in prevention initiatives. This effect seems to exist also in parents with a migration background and a high level of education.

## Strengths and limitations

This is one of the few studies on obesity focussing on the transitional phase from kindergarten to primary school and to investigate the importance of neighbourhood deprivation on the basis of a large sample with low selection bias [42]. One advantage is that we were able to draw upon medically determined body weights. In contrast, a number of studies used subjective information which raises the problem of incorrect memories or social desirability. A disadvantage is, however, that we have measured the individual SEP only on the basis of parental education. This produces missing values and the prevalence of children with obesity, living in deprived neighbourhoods and with low and medium educated parents is underestimated. Sensitivity analyses were conducted to test the robustness of findings with individual income as another indicator of individual SEP. Due to missing values on income (15%) and household size (32%), the calculation of the net equivalent income was only possible using a much smaller sample (4318 children). Analogous to the study by Rossen [33], the relationship between neighbourhood deprivation and child obesity was stronger for higher parental income compared to lower parental income; however, the interaction terms were not significant. This could be due to the small sample of obese children (*n*=393), resulting in small numbers for the categories. Finally, with the existing data, neighbourhood environment is measured by socioeconomic aspects, only. As we have pointed out at the beginning, these influence the spatial environment such as walkability and green spaces. Based on linkage with community geo data, this should be subject to further research.

## Conclusions

In regard to child obesity prevention, there is a need to take action in deprived neighbourhoods. Through small-scale analysis of social structures particularly deprived urban neighbourhoods can be identified. The precise mechanisms through which the neighbourhood influences childhood development have not been clarified [38] nor have sufficient evidence been provided on the effectiveness of interventions [4]. Nonetheless, there are many recommendations on policies and environmental interventions [5, 11, 29, 41] including school environments [28].

Second, action is needed regarding children of parents with low education. Health literacy could be a field for intervention here, as it is less in low education groups [40]. Parental health literacy is associated with attitudes towards weight control for children [21] and child obesity [6, 25]. Consequently, strategies for weight loss in children should aim towards strengthening the health literacy of parents and supporting them in accessing evidence-based information [6, 21]. However, such approaches should be culturally sensitive [32].

## Data Availability

Data and material are not available for third parties.

## Abbreviations

BMI: Body mass index
CASMIN: Comparative analysis of social mobility in industrial nations
CI: Confidence interval
PR: Prevalence ratio
SD: Standard deviation
SEP: Socioeconomic position
VIF: Variance inflation factor

## Code availability

All analyses were conducted using Stata 14.
